# A Rapid and Noninvasive Method That Extracts Polymerase Chain Reaction-Ready Genomic DNA from Adult Zebrafish

**DOI:** 10.1089/zeb.2022.0015

**Published:** 2022-08-11

**Authors:** Ji Yun Jang, Ting Liang, Myeong-Kyu Kim, Kyung Wook Kang, Bora Lee, Seok-Yong Choi

**Affiliations:** ^1^ Department of Biomedical Sciences, Chonnam National University Medical School, Hwasun, Republic of Korea.; ^2^ Department of Neurology, Chonnam National University Medical School, Gwangju, Republic of Korea.; ^3^ Department of Neurology, Chonnam National University Hospital, Gwangju, Republic of Korea.; ^4^ Department of Biochemistry, Chonnam National University Medical School, Hwasun, Republic of Korea.

**Keywords:** extraction, genomic DNA, genotyping, Q-tips, swabbing, zebrafish

## Abstract

Genotyping usually entails analysis of the products of polymerase chain reaction (PCR) carried out with genomic DNA (gDNA) as template, and is employed for validation of mutant or transgenic organisms. For genotyping of adult zebrafish, gDNA is often extracted from clipped caudal fin or skin mucus through either alkaline lysis using NaOH or proteinase K (PK) treatment. Further purification of the gDNA using ethanol precipitation was optional. To develop a rapid and noninvasive method that extracts PCR-ready gDNA from adult zebrafish, we combined skin swabbing with PK treatment and demonstrated its efficiency. This method could be applied to a wide range of fish.

## Introduction

Zebrafish is a popular model organism in life science research due to its high fecundity, external and visually accessible development, and amenability to various genetic manipulations including *N*-ethyl-*N*-nitrosourea- or clustered regularly interspaced short palindromic repeats (CRISPR)/CRISPR-associated protein 9 (Cas9)-mediated mutagenesis and transgenesis.^1^ Validation of mutant or transgenic zebrafish often requires genotyping, which usually entails analysis of products of polymerase chain reaction (PCR) carried out with genomic DNA (gDNA) as the template DNA.^2^

Zebrafish gDNA can be extracted from several sources: whole organisms are used when extracted from embryos, whereas tail fins or skin mucus are utilized in adult zebrafish. Tail fins have been a preferred source of adult zebrafish gDNA because they can regenerate after cutting (clipping).^3^ However, fin clipping is an invasive method that may lead to pain, stress, or infection. As such, procurement of zebrafish skin mucus using cotton swabs (Q-tips^®^), called “skin swabbing,” was presented as an alternative noninvasive method.^4^

Once zebrafish tissue or mucus is obtained, either the alkaline lysis method using NaOH or the proteinase K (PK) method is employed to extract gDNA. However, both methods have disadvantages. As NaOH is very corrosive, it has to be neutralized with Tris buffer.^5^ As residual PK may inhibit subsequent PCR, it should be heat inactivated before PCR.^6^ In general, gDNA extraction using NaOH, although popular, takes longer than through PK treatment.

The resulting extracted gDNA can be purified further using either the ethanol precipitation method or commercial DNA binding columns. However, this purification step is usually not required for PCR-mediated genotyping.^7^

To establish a rapid and noninvasive method for extraction of PCR-ready gDNA from adult zebrafish, we combined swabbing of the skin mucus with PK-mediated gDNA extraction and tested the efficiency of this method.

## Materials and Methods

### Fish husbandry

Wild-type zebrafish (AB strain) and *mitopld* mutant zebrafish were obtained from the Zebrafish International Resource Center and Fluorescent Reporter Zebrafish Cooperation Center (no. 1120), respectively, and maintained using standard procedures described previously.^7^

### Fin clipping and extraction of gDNA

Caudal fin of zebrafish was clipped as described previously.^8^ The clipped fin was placed in a microfuge tube containing 50 μL of 50 mM NaOH, incubated at 95°C for 10 min followed by at 4°C for 10 min, neutralized with 5 μL of Tris HCl (pH 9.5), and centrifuged at 16,000*g* at room temperature for 10 min in a bench-top centrifuge (5415R; Eppendorf). DNA concentration was measured using a UV spectrophotometer (BioSpectrometer; Eppendorf).

### Skin swabbing and extraction of gDNA

**FIG. 1.**
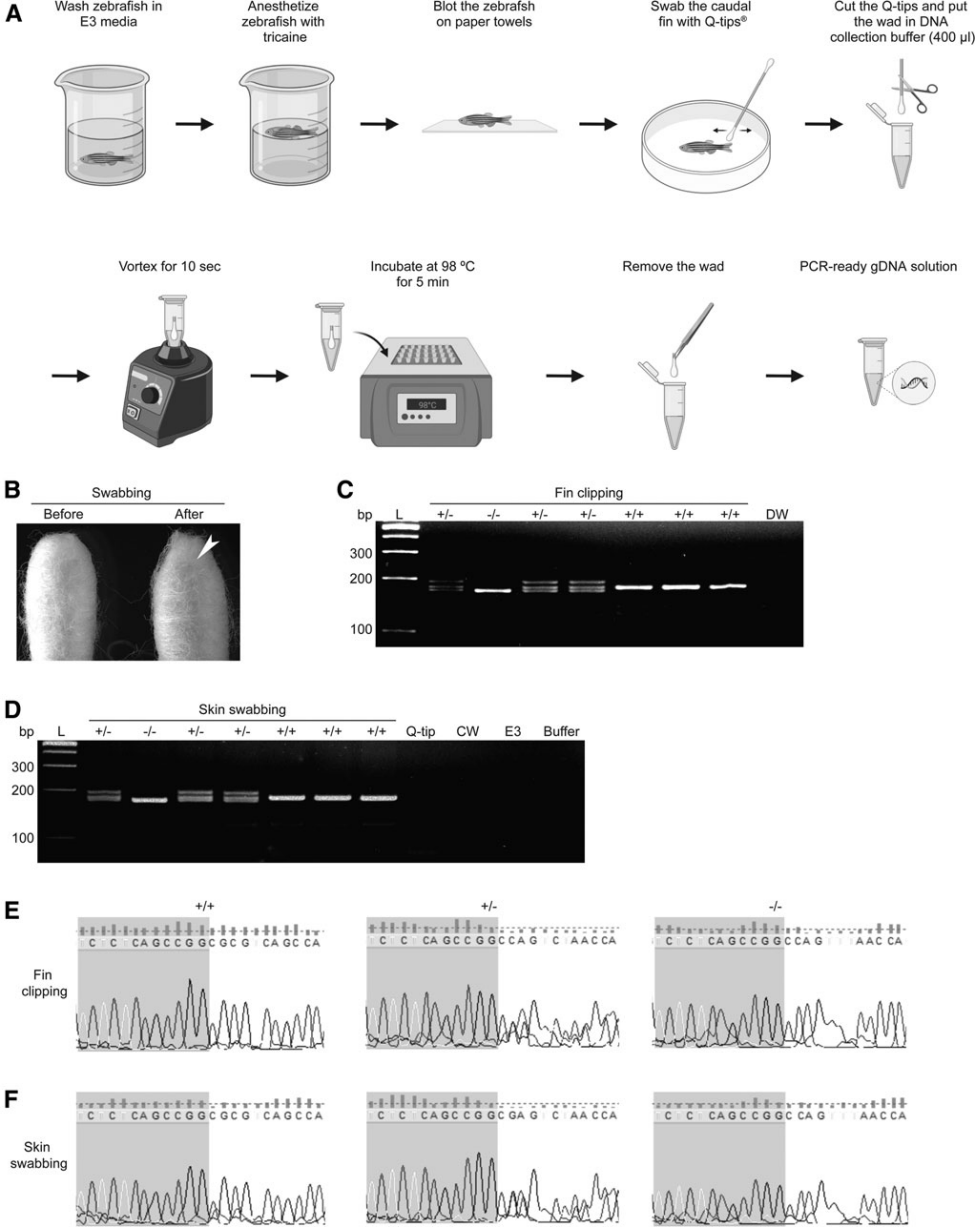
**(A)** Schematic overview depicting a rapid and noninvasive method for extracting PCR-ready gDNA from adult zebrafish. Generated with BioRender. **(B)** Light microscopic images of cotton swabs before and after swabbing. *Arrow* indicates mucus on the wad. **(C, D)** *mitopld* was PCR amplified from gDNA extracted from clipped fin **(C)** or swabbed cotton wad **(D)**, and 4 μL **(C)** and 8 μL **(D)** of PCRs were then analyzed using 4% agarose gel electrophoresis. **(E, F)** Electropherograms of PCR products in **(C)** and **(D)**, respectively. +/+, WT *mitopld*; +/−, heterozygous *mitopld* mutant; −/−, homozygous *mitopld* mutant (8 bp deletion); bp, base pairs; buffer, DNA collection buffer; CW, cage water; DW, distilled water; E3, E3 embryo medium; gDNA, genomic DNA; L, DNA ladder; PCR, polymerase chain reaction; Q-tip, clean Q-tip; WT, wild-type.

Zebrafish at 2 months postfertilization (∼25 mm in length) was transferred to a mating cage containing E3 embryo medium (5 mM NaCl, 0.17 mM KCl, 0.33 mM CaCl_2_, and 0.33 mM MgSO_4_), rinsed, anesthetized for 1 min with 1.5 mg/mL tricaine (ethyl 3-aminobenzoate methanesulfonate salt; E10521; Sigma-Aldrich) in E3 embryo medium, blotted on paper towels to remove excess water, and then placed in a petri dish (90 × 15 mm, 10093, SPL Life Sciences; Fig. 1A).

The zebrafish was swabbed several times on the caudal fin with a cotton swab (Q-tips^®^ Cotton Swabs) until the tip of the swab became yellowish (Fig. 1B). During swabbing, contact with the trunk region was avoided to prevent potential injury to the internal organs. Of note, zebrafish should be at least 20 mm in length to keep them alive after the swabbing (Supplementary Fig. S1).

Subsequently, the cotton swab was cut 1 cm above the cotton wad, which was then put in a microfuge tube harboring 400 μL of DNA collection buffer (30 mM Tris-HCl, and 1 μg/μL PK [03115828001; Roche]). The microfuge tube was vortexed for 10 s and incubated at 98°C for 5 min. The cotton wad was removed and the resulting solution was used for subsequent PCR.

### PCR and DNA sequencing

The extracted gDNA was subjected to PCR in a 10 μL reaction: 0.1 μL Taq DNA polymerase (G-1000; GENETBIO), 5 pM of each gene-specific primer, 1 mM dNTP, and 1 μL reaction buffer. The primer pairs used to PCR-amplify the zebrafish *connexin43 (cx43)* and zebrafish *mitopld (*also called *pld6)* were 5′-CGCACCTACATCTTCAGCATCAT-3′ (forward) and 5′-ACGCGGTCCTTGATTCGTTTGA-3′ (reverse), and 5′-GTCATTTAAGGAGCTGATG-3′ (forward) and 5′-AGCTTCGGTGAGATGTGAAC-3′ (reverse), respectively. The resulting PCR products were analyzed with 4% agarose gel electrophoresis and sequenced at Cosmo Genetech.

## Results and Discussion

gDNA was extracted from the clipped zebrafish fin or swabbed mucus. The concentration of gDNA obtained by skin swabbing was 31.68 ± 3.64 ng/μL (mean ± standard deviation; A_260_/A_280_ = 1.25 ± 0.09; *n* = 5 hereafter), which is lower than that obtained by fin clipping (130.60 ± 33.57 ng/μL; A_260_/A_280_ = 1.49 ± 0.06). This is not surprising as the number of cells is expected to be higher in the clipped fin than in the swabbed mucus.

To test the quality of the extracted gDNA, we performed PCR and sequenced its products. *mitopld* was well amplified, showing that the gDNA extracted by skin swabbing is indeed PCR ready. No PCR product was noted from cage water, distilled water, DNA collection buffer, E3 embryonic medium, or clean Q-tip, ruling out contamination of water, buffer, or Q-tip. Of note, heteroduplex formation (double bands) indicates the heterozygous mutant of *mitopld* (Fig. 1C–E).

The skin swabbing method reported by Breacker *et al.* employed isopropanol precipitation.^4^ Therefore, their isolated gDNA was pure (A_260/280_ = 1.99 ± 0.09), yet it took >50 min to complete the Breacker protocol on our hands. In contrast, our isolated gDNA was not pure (A_260_/A_280_ = 1.25 ± 0.09), yet this was still sufficient for further PCR. Moreover, it took only 10 min to complete our protocol.

In this study, we demonstrate that PK-mediated gDNA extraction from swabbed skin mucus is a rapid, noninvasive, and reliable method that can extract PCR-ready gDNA from adult zebrafish. When held at high density, mucus transfer might occur among zebrafish, leading to cross-contamination. However, skin swabbing followed by PCR did not reveal any cross-contamination among zebrafish raised at high density.^4^ Furthermore, skin swabbing was reported to induce less stress axis activation and fewer changes in behavior and physiology than fin clipping.^9^ Finally, skin swabbing followed by PK-mediated gDNA extraction could be easily applied to a wide range of fish species.

## Supplementary Material

Supplemental data
