# Supplementary material for: A Rapid and Noninvasive Method That Extracts Polymerase Chain Reaction-Ready Genomic DNA from Adult Zebrafish
Source: Zebrafish. 2022 Aug 11;19(4):160–4. doi: 10.1089/zeb.2022.0015 (PMC9419989; doi:10.1089/zeb.2022.0015)
Supplement: Supplemental data [file Supp_FigS1.pdf]

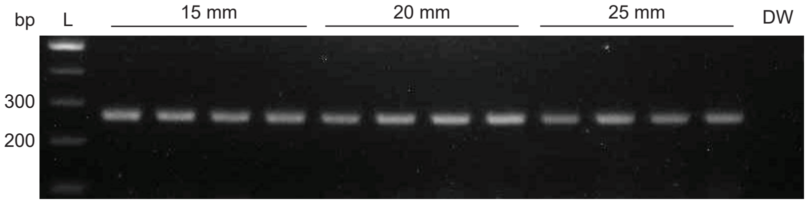

Supplementary Figure. *connexin43* (*cx43*) was PCR-amplified from genomic DNA extracted from zebrafish with indicated size in length and then analyzed using agarose gel electrophoresis. bp, base pairs; DW, distilled water; L, DNA ladder.
